# START NOW WebApp—promoting emotion regulation and resilience in residential youth care and correctional institutions: study protocol for a cluster randomized controlled trial

**DOI:** 10.1186/s13063-024-08180-z

**Published:** 2024-05-22

**Authors:** Linda Kersten, Janine Alfano, Tobias E. Erlanger, Fabrice Helfenstein, Lelia Lanz, Stefan Weiss, Chiara Chilla, Beryll von Planta, Madlaina Kapoor, Nathalie Borel, Tabea Rocco, Andreas Papageorgiou, Catarina Fernandes De Brito, Arzie Bajrami, Valentine Savary, Melanie Mayor, Jana Hurschler, Alex Traut, Donja Brunner, Noortje Vriends, Christina Stadler

**Affiliations:** 1 Department of Child and Adolescent Psychiatry, University Psychiatric Clinics Basel, University of Basel, Wilhelm Klein-Strasse 27, 4002 Basel, Switzerland; 2 Department of Clinical Research, University of Basel and University Hospital Basel, Basel, Switzerland

**Keywords:** Randomized controlled trial, Intervention, Adolescents, Young adults, Residential youth care, Emotion regulation, Psychological flexibility, Resilience

## Abstract

**Background:**

Adolescents and young adults in residential care and correctional institutions face various challenges, leading to negative life outcomes. Implementation barriers within these institutions, such as limited financial and spatial resources, pose significant hurdles to providing necessary support. Web-based approaches address these challenges by offering cost-effective, accessible solutions. This study aims to assess the efficacy of a newly developed web-based version of the existing evidence-based START NOW skills training in fostering emotion regulation and resilience among institutionalized adolescents and young adults. We present the study protocol (Version 5, August 2023) of the trial titled “Implementation of an e-version of the skills training START NOW for promoting emotion regulation and resilience in residential youth care and correctional institutions”.

**Methods:**

The study is a monocentric, prospective, confirmatory randomized controlled trial with 150 institutionalized adolescents and young adults with a need to improve resilience (predefined cut-offs). Participating institutions will be randomized to one of three conditions: (i) 9-week web-based group training guided by a facilitator, (ii) 9-week web-based self-help training, (iii) and treatment as usual. The primary endpoint is the change in psychological flexibility, assessed by the Avoidance and Fusion Questionnaire for Youth score, from baseline to follow-up 12 weeks post skills training. Secondary objectives encompass assessing pre-post changes in psychological flexibility and other psychological health-related outcome measures in participating adolescents, young adults, and caretakers from baseline, to post training, and to 12- and 24-week follow-ups.

**Discussion:**

This study evaluates the efficacy of START NOW as web-based training for institutionalized adolescents and young adults, providing valuable insights into web-based interventions and aiming to optimize support levels.

**Trial registration {2a and 2b}:**

ClinicalTrials.gov NCT05313581. Registered on 6 April 2022.

**Supplementary Information:**

The online version contains supplementary material available at 10.1186/s13063-024-08180-z.

## Introduction

### Background and rationale {6a}

Numerous studies on adolescents and young adults (AYA) indicate a much higher rate of mental health problems in residential youth care (RYC) and correctional institutions (CI) than in the general population [1, 2]. The presence of risk factors (such as impulsivity, no family/social support, emotion regulation deficits) further exacerbates an AYA’s risk for negative life outcomes [3, 4] with staggering costs to society [5, 6]. In general, the burden in affected children and AYA is extremely high, emphasizing the need for innovative, tailored approaches to bring evidence-based care to settings with limited resources [7–10].

START NOW is one integrative approach well-suited for settings with resource constraints and an extensive need for effective, reliable treatment for individuals exhibiting impairments in emotion regulation and management of social competencies [11]. The intervention comprises a manualized skills training with mindfulness exercises, functional analyses of emotion and behavior, as well as, specific topics such as accepting emotions, building up interpersonal skills, and setting goals, thus promoting general psychological health and resilience. START NOW facilitators undergo a training providing background information on basic aspects of cognitive behavioral therapy (CBT), motivational interviewing (MI), dialectical behavior therapy (DBT), acceptance and commitment therapy (ACT), and trauma-sensitive care, as well as practical exercises on how to facilitate the skills training [12]. There is accumulated support for the use of the START NOW intervention with adult forensic and correctional populations, institutionalized AYA as well as student populations. Indeed, studies have shown START NOW’s effectiveness in terms of reduced behavioral problems [13], reduced hospitalization rates [14], improved mental health functioning [15] and high satisfaction rates [16]. In addition, Stadler et al. [17] adapted START NOW for AYA and investigated its implementation and efficacy within an international randomized trial. Results demonstrated START NOW`s effectiveness in reducing aggressive and oppositional behaviors in institutionalized AYA.

However, challenges persist in implementing skills training programs in RYC and CI, such as institutional or personal constraints (staff shortage, high staff turnovers, working in shifts), as well as constraints in AYA (limited time or motivation, discharge/transfer of institution), ultimately limiting the effectiveness of such programs [18, 19]. Web-based approaches, delivering health services on various devices, offer promising solutions to outlined implementation barriers, providing cost-effectiveness, high accessibility, flexibility, direct resource utilization, anonymity, reduced stigma, and a practical means for ensuring continuity of care during transitional phases [20, 21].

The effectiveness of web-based health approaches is well established across settings, populations (e.g., non-clinical, clinical, adolescents, adults), and a range of mental health outcomes, such as improvement of clinical symptoms, mindfulness, and quality of life/well-being [22–25]. Studies directly comparing the efficacy of face-to-face and web-based approaches show no significant differences between the two approaches [26–28]. Yet, web-based approaches can vary substantially in their delivery format ranging from unguided self-help services to guided services (human support mostly through tailored, written feedback) to real-time, direct human support through, for example, videoconferencing or telephone services. With regard to specific delivery format, the efficacy of web-based approaches increases with the extent of support: Web-based approaches including human support come with larger effects and greater adherence compared to unguided self-help and tend to be as effective as face-to-face intervention formats [29–32].

Thus, the current study aims to investigate whether a web-based version of START NOW—with or without additional support—is effective in enhancing psychological flexibility (PF), a core indicator of good mental health and resilience [33–36]. Additionally, through qualitative interviews, we will investigate whether web-based applications are suitable for overcoming given constraints, consequently, contributing to a better and more sustainable implementation of evidence-based care in RYC and CI.

### Objectives {7}

This project aims to evaluate a web-based translation of the existing START NOW skills training that promotes resilience and emotion regulation, operationalized through PF, in institutionalized AYA, while addressing the numerous barriers to program implementation in the RYC and CI settings. We will investigate the efficacy of a web-based version of START NOW on PF in a randomized controlled trial comparing the following conditions: (1) web-based group training guided by a facilitator (trained START NOW facilitator, providing guidance through sessions and motivating change), (2) web-based self-help training (without guidance, with automated feedback responses), and (3) treatment as usual (TAU). In accordance with the literature, we expect to see the largest effect on PF within the web-based group training guided by a facilitator. PF and respective inflexibility will be assessed by the change in self-rated Avoidance and Fusion Questionnaire for Youth (AFQ-Y) [37] score from baseline to follow-up (12 weeks post skills training).

#### Hypotheses

1. In institutionalized AYA web-based START NOW skills training is more efficient when delivered as group training guided by a facilitator (motivates to change behavior) than TAU in decreasing psychological inflexibility (primary outcome) as measured by AFQ-Y scores 12 weeks ± 2 weeks (T_3_) after the end of the intervention.

Statistical hypothesis:

- H_0_: 12 weeks after the end of the intervention (at T_3_), mean inflexibility, as assessed by the Avoidance and Fusion Questionnaire for Youth (AFQ-Y), is not different in participants receiving TAU and in participants receiving a web-based START NOW training.
- H_a_: 12 weeks after the end of the intervention (at T_3_), mean inflexibility, as assessed by the Avoidance and Fusion Questionnaire for Youth (AFQ-Y), is different in participants receiving TAU and in participants receiving a web-based START NOW training.

2) In institutionalized AYA START NOW skills training is more efficient when delivered as web-based self-help training than TAU in decreasing psychological inflexibility (primary outcome) as measured by AFQ-Y scores 12 weeks ± 2 weeks (T_3_) after the end of the intervention.

#### Secondary objectives

The skills training is expected to improve general resilience and decrease depression and anxiety symptoms, and irritability [13, 17]. Building on this, the START NOW skills training in a group guided by a facilitator may be more effective than self-help web-based training and TAU in enhancing resilience, self-efficacy, well-being, and reducing impairment, depression, anxiety, and irritability. In addition, different covariates and potential moderators including gender, age, access to the internet, caretaker/facilitator, or institutional criteria will be investigated. A positive social climate has been associated with positive outcomes, such as motivation for the intervention, active use of skills, greater intervention satisfaction and resilience, and fewer problem behaviors, such as bullying, aggression, or social problems [38]. Therefore, the moderating effect of the social atmosphere within the institution will also be examined. Former positive feedback from personnel trained in START NOW [17] lead to the hypothesis, that caretakers completing START NOW facilitation courses will exhibit improved resilience, with greater gains in the group training condition compared to self-help and TAU. Gains are linked to better insights, improved self-efficacy, and more opportunity for positive exchanges with youth. The caretakers’ level of resilience, as caretakers in the active intervention conditions will partake in a START NOW training course, will be examined in the study. Furthermore, participants’ and caretakers’ satisfaction with the training will be evaluated.

### Trial design {8}

The study is a monocentric, prospective, confirmatory randomized controlled trial with three experimental conditions: (1) START NOW as web-based group training guided by a facilitator, (2) START NOW as web-based pure self-help training, and (3) TAU. Institutions are randomly assigned 2 weeks before the 9-week intervention phase.

## Methods: participants, interventions, and outcomes

### Study setting {9}

The study is conducted by the Department of Child and Adolescent Psychiatry, University Psychiatric Clinics Basel. Data is collected in RYC a CI recognized by the Federal Office of Justice (FOJ) [39] throughout the German and French-speaking parts of Switzerland. Assessments are conducted at five time-points (T). First to screen for eligibility (T_0_), then before intervention start (T_1_), immediately following intervention end (T_2_), 12 weeks ± 2 weeks after intervention end (T_3_), and 24 weeks ± 2 weeks after intervention end (T_4_).

### Eligibility criteria {10}

Inclusion criteria are listed in Table 1.

**Table 1:** Eligibility criteria for institutions and participants

Inclusion criteria for institutions:	Inclusion criteria for participants:
• Institutional consent • Ability to offer a 9-week group training guided by a facilitator • At least two designated START NOW trainers that successfully complete the full 1.5 days of training • Internet access for participants • Guarantee of standardized implementation	• AYA in RYC or CI • Ages 14 to 24 years • Sufficient German or French language skills • Participant consent • AFQ-Y sum score ≥ 34.05 • MAYSI-2 subscale angry-irritable sum score ≥ 5.0 • MAYSI-2 subscale depressed-anxious sum score ≥ 3.0 • No concurrent group-based skills training

*Note. AYA* adolescents and young adults*; AFQ-Y* Avoidance and Fusion Questionnaire for Youth; *MAYSI-2* Massachusetts Youth Screening Instrument-2. Participants with high scores on MAYSI-2 subscales Suicide-Ideation (≥ 2) or Thought-Disturbance (≥ 2) are individually assessed with caretakers. Suicidal or otherwise acutely endangered participants are excluded

### Consent or assent {26}

#### Who will take informed consent? {26a}

A trained member of the START NOW study team provides information about the study, including study procedures and informed consent, to all interested participants (in-person or online). Consenting AYA fill out a physical consent form. Forms are checked by the research team and filed in a secure location before participation is approved.

#### Additional consent provisions for collection and use of participant data and biological specimens {26b}

Not applicable, as no biological specimens are collected in this study.

### Interventions {11}

#### Explanation for the choice of comparators {6b}

We will evaluate the efficacy of web-based START NOW on PF in a randomized controlled trial with three conditions: (1) web-based group training guided by a facilitator, (2) web-based self-help training, and (3) TAU. The inclusion of guided group training aims to assess the benefits of facilitator support, examining its potential superiority in delivering START NOW. Incorporating a self-help approach considers the resource implications of human-supported interventions, exploring the standalone effectiveness of START NOW. TAU acts as a control condition to isolate and assess specific impacts of the START NOW Web Application (WebApp). In summary, our chosen comparators systematically assess the effectiveness of web-based START NOW, considering human support advantages, resource implications, and the need for a relevant control group.

#### Intervention description {11a}

The WebApp is based on the manualized START NOW skills training. Each session is structured and comprises mindfulness exercises, functional analyses of emotion and behavior, as well as specific topics such as accepting emotions, building up interpersonal skills, and setting goals. Content is available in German and French across 12 sessions, incorporating gender-inclusive elements. It employs comics, video clips, and various exercises. Participants can track their progress, collect trophies, and have the option to become co-trainers by the end of the intervention. The WebApp aligns with Good Clinical Practice Guidelines and was validated by the Department of Clinical Research (DCR), University of Basel, and University Hospital Basel. Throughout the 9-week intervention phase, START NOW sessions unlock weekly with sessions 1 and 2, 9 and 10, and 11 and 12 being double sessions. The sessions are conducted either a) as 12 web-based group trainings (4 to 12 participants) guided by a facilitator (face-to-face or videoconferencing; duration 60 min; 120 min for double sessions), or b) as self-help training (duration 45 +/- 15 min; 90, +/− 15 min for double sessions). All participants have access to the START NOW WebApp during the entire intervention and follow-up phase to complete additional exercises or review content. Sessions will be unlocked on a weekly basis to keep the schedule between conditions consistent. Participants will receive reminders about unlocked sessions, though they may ignore the overall session order and may finish unlocked sessions out of order. Additionally, participants will be able to repeat content of previous sessions, to do bonus assignments for themselves, and to contribute to a forum. Participants of the TAU conditions will be provided with web-based self-help training after completion of the study. Facilitation will be provided either by a trained staff member (caretaker) of the institution or by a member of the START NOW facilitator team of the University Psychiatric Clinics (UPC) Basel (videoconference setting). The 12 h START NOW training for RYC/CI staff members covers session content and facilitation and includes background information on the core elements of the intervention: MI, CBT, DBT, and trauma-sensitive care.

#### Criteria for discontinuing or modifying allocated interventions {11b}

Any participant can withdraw their consent for the study at any time. The principal investigator and the Basel study team can also decide about termination of intervention for individual participants: in case of adverse events (AE), or if, in the investigators’ opinion, continuation in the investigation would be detrimental to the participants’ well-being. The date of withdrawal is to be documented. The Ethics Committee (EC) and the competent authorities must be informed about premature closure of the trial or one of the intervention arms. Furthermore, the EC(s) and competent authorities themselves may decide to stop or suspend the trial.

#### Strategies to improve adherence to interventions {11c}

To ensure standardized implementation by facilitators, participating staff will be adequately trained. Also, the quality of the group training guided by a facilitator will be rated by a research team member during the second session (either live or via online connection) using a Quality Assurance Form [40]. Supervision for participating caretakers and facilitators (except TAU condition) will be provided twice during the intervention phase: First time immediately after the second session, the second time in the first half of the intervention (2 h per session). Moreover, caretakers and facilitators can reach out via e-mail to assigned START NOW study coordinators for further guidance. The purpose is to assist caretakers and facilitators in the realization of START NOW, to cope with difficult situations, to progress in expertise, as well as to ensure good service to the participants. After every session of the group training, attendance of the participants will be assessed via e-mail or phone with the responsible trainer.

Participants will be compensated with online shopping vouchers for participating in questionnaires (CHF 20 for one completed time-point). They can additionally earn a special co-trainer certification after completing the full training. Additionally, all web-based training aspects allow collection of virtual coins, medals, or diamonds (depending on the activity) which are displayed on a progress screen. Participants are also reminded regularly about new and not yet finished sessions.

The material also highlights the potential benefits for participants, addressing the possibility of better stress and emotion management and encouraging self-efficacy.

#### Relevant concomitant care permitted or prohibited during the trial {11d}

Relevant additional treatments (e.g., individual psychotherapy) administered to the participant upon entry to the trial or at any time during trial participation are regarded as concomitant treatments and will be documented. Concurrent participation in CBT-based skills trainings similar to START NOW disqualifies from participation. Concomitant medication will be recorded.

### Provisions for post-trial care {30}

Not applicable. The study has undergone evaluation by the Ethics Commission Northwestern and Central Switzerland (EKNZ) and has been categorized as having minimal risks and burdens (Risk category A according to ClinO, Article 61). The study does not involve any invasive or clinical data assessment procedures that would cause adverse (psychological) responses.

### Outcomes {12}

#### Primary endpoints

The primary focus is on the post-intervention psychological inflexibility measured by AFQ-Y scores between baseline (T_1_) and follow-up (T_3_) (12 weeks +/− 2 weeks after end of intervention). The AFQ-Y is a validated self-rating questionnaire assessing PF in adolescents and young adults. Items are based on ACT models of human suffering representing the theoretical concept of psychological inflexibility due to high cognitive fusion and experiential avoidance [37]. Participants answer 17 items indicating how true each item is for them on a 5-point Likert scale (0 = not at all true; 4 = very true). Higher total scores indicate lower PF. Data will be assessed within 2 weeks before start of skills training (baseline, T_1_), within 2 weeks after end of skills training (T_2_), as well as at 12 weeks (+/− 2 weeks) (T_3_) and 24 weeks (+/− 2 weeks) (T_4_) post skills training. Primary endpoint is the change in total score between baseline (T_1_) and follow-up (T_3_). Total scores can range between 0 and 68. Accordingly, a change in score can range between − 68 and + 68.

Further objectives of the current trial are to assess the effect of treatment (immediately after the end of intervention) on other psychological health-related outcome measures (i.e., general resilience, psychological well-being, self-efficacy, general impairment, anger-irritability, training substance use) in participants of the three conditions. Furthermore, resilience will also be assessed for caretakers.

#### Secondary endpoints

Change will be assessed at baseline (T_1_) and (i) post skills training (T_2_), (ii) 12-week follow-up (T_3_), and (iii) 24-week follow-up (T_4_).

- Resilience: *Connor-Davidson Resilience Scale* (CD-RISC) [41] Self-reported psychological well-being: *World Health Organization - Five Well-Being Index* (WHO-5) [42] in self-rating by participant
- Self-reported self-efficacy: total score on the German *Skala zur Allgemeinen Selbstwirksamkeitserwartung* [*General Self-Efficacy Scale]* (SWE) [43] in self-rating by participant
- General impairment: all sub-scores and total score on the *Columbia Impairment Scale* (CIS) [44] both in self-rating by participant and external rating by caretaker
- Depression and anxiety: total score on the *Patient Health Questionnaire-4* (PHQ-4) [45, 46] in self-rating by participants
- Anger-irritability: total score on *Affective Reactivity Scale* (ARI) [47] both in self-rating by participant and external rating by caretaker
- Substance use: total score on the Alcohol/Drug Use subscale of the *Massachusetts Youth Screening Instrument-2* (MAYSI-2) [48] in self-rating by participants

##### Resilience

General resilience will be measured by the *Connor-Davidson Resilience Scale* (CD-RISC) in the 10-item version [49]. It refers to an individual’s ability to endure difficult experiences. This scale consists of 15 items on a 5-point Likert scale ranging from 1 (strongly disagree) to 5 (strongly agree). Total scores can range from 0 to 50, with change scores ranging from − 50 to +50.

##### Well-being

Psychological well-being will be assessed by *The World Health Organisation- Five Well-Being Index* (WHO-5) [42]. The self-report questionnaire contains five items on a 6-point Likert scale ranging from 5 (*all of the time)* to 0 *(none of the time*). Respondents are asked to indicate how often they felt well during the last 2 weeks. Total scores can range from 0 to 25, with higher scores indicating greater well-being. Accordingly, change scores range from − 25 to +25.

##### Self-efficacy

Self-efficacy will be assessed by the German self-report questionnaire *Skala zur Allgemeinen Selbstwirksamkeitserwartung [General Self-Efficacy Scale]* (SWE) [43]. The questionnaire includes 10 items and a 4-point Likert scale ranging from 1 *(not true) to 4 (completely true*). The scale reflects one’s convictions on subjective controllability or competence expectations in different demanding situations, with higher scores indicating a greater sense of self-efficacy. Total scores can range from 10 to 40, with change scores ranging from − 30 to +30.

##### General impairment

General impairment will be assessed by the *Columbia Impairment Scale* (CIS) [44]. The 13-item questionnaire captures functional impairment in four domains: interpersonal relations, broad psychopathological domains, functioning in school or at work, and use of leisure time. It will be answered both by the participant as a self-report questionnaire, and by the caretakers as external raters. Items are answered on a 5-point Likert scale ranging from 0 (*no problem) to* 4 (a *very big problem*). Total score can range from 0 to 52, with change scores ranging from − 52 to +52.

##### Depression and anxiety symptoms

The Patient Health Questionnaire-4 (PHQ-4) is a short questionnaire with two sub-scales (depression and anxiety). The PHQ-4 is a reliable, valid, and precise screening tool for self-reported depression, anxiety, and general psychological distress [45, 46, 50]. Answers are given on a 4-point Likert scale ranging from 0 (not at all) to 3 (nearly every day). The sum of all items comprises the total score. Total scores are rated categorized as normal (0 to 2), mild (3 to 5), moderate (6 to 8), and severe (9 to 12). A score ≥ 3 for items 1 and 2 suggests anxiety. A score ≥ 3 for items 3 and 4 suggests depression.

##### Anger-irritability

Reactivity will be measured by the *Affective Reactivity Index* (ARI) [47]. It will be answered by AYA youth as self-report questionnaire and by caregivers as external raters to indicate irritable mood. It contains seven items to be scored on a 3-point Likert scale ranging from 0 (*not true) to 2 (certainly true*). The total score is calculated as the sum of items 1 to 6, resulting in a range from 0 to 12. Item 7 addresses the perceived degree of suffering and is analyzed separately.

##### Substance use

Within the screening for eligibility, signs of mental/emotional disturbance, such as alcohol and drug use, will be assessed using the *Massachusetts Youth Screening Instrument-2* [48]. It is a brief screening tool designed for adolescents between the ages of 12 to 17 years. The MAISY-2 contains 52 items across seven subscales: alcohol/drug use, anger-irritability, depression-anxiety, somatic complaints, suicide ideation, traumatic experiences, and thought disturbance. Respondents are asked about the presence of various thoughts, feelings or behaviors in the past few months, in a *yes* or *no* format. Each subscale contains different caution cut-offs.

##### Client training satisfaction

Participants within the two intervention conditions can indicate their satisfaction with the training using the *Client Satisfaction Questionnaire* (CSQ) [51] at T_2_ (post intervention). The CSQ was specifically developed to assess participants’ experiences and satisfaction with START NOW. This scale consists of 8 items on a 4-point Likert scale ranging from 0 to 3 and two open questions about what participants liked or would want to be changed within the training. Total scores can range from 0 to 24.

##### Trainer satisfaction

Satisfaction of facilitators within the guided group training condition will be assessed with the *Trainer Satisfaction Questionnaire* (TSQ) [51] at T_2_ (post intervention), a 5-min self-rating questionnaire. After the conclusion of the second follow-up (T_4_), individual semi-structured expert interviews are planned for all interested institutions, whether they participated in the study or were unable to do so (minimum of two individually interviewed employees per institution). The interviews will last 45–60 min and cover topics such as reach, effectiveness, adoption, implementation, and maintenance of intervention programs, including START NOW.

##### Social atmosphere

Social atmosphere will be assessed with the German version of the *Essen Climate Evaluation Schema* (EssenCes) [52]. It is a short self-rating questionnaire, containing 17 items across three subscales: therapeutic hold, patients’ cohesion and mutual support, and experienced safety. Responses are given on a 5-point Likert scale ranging from 0 (*not at all)* to 4 *(very much*). Total scores can range from 0 to 68 with higher scores indicating a better social climate.

##### Checklist baseline T_1_ and checklist monitoring

Inclusion/exclusion criteria throughout the study (i.e., participation in concurrent CBT-based skills training similar to START NOW., internet access, external placements/discharge, unauthorized leaves) will be screened/monitored with two checklists specially designed for this purpose.

##### Prior experiences of caretakers and facilitators

The professional qualification of involved caretakers and facilitators and their experiences with START NOW or other forms of resilience trainings will be assessed with a short questionnaire. For caretakers, the assessment is at baseline (T_1_), for facilitators, professional qualification is assessed post-intervention (T_2_).

##### Adherence

For each institution, the quality of the group training guided by a facilitator will be rated by a START NOW facilitator during the second session (either live or via online connection) using a *Quality Assurance Form* [40]. Furthermore, participant adherence will be recorded through a) attendance lists created by caregivers in the group condition and b) usage logs of the WebApp, specifically finished sessions.

### Participant timeline {13}

**Fig. 1:**
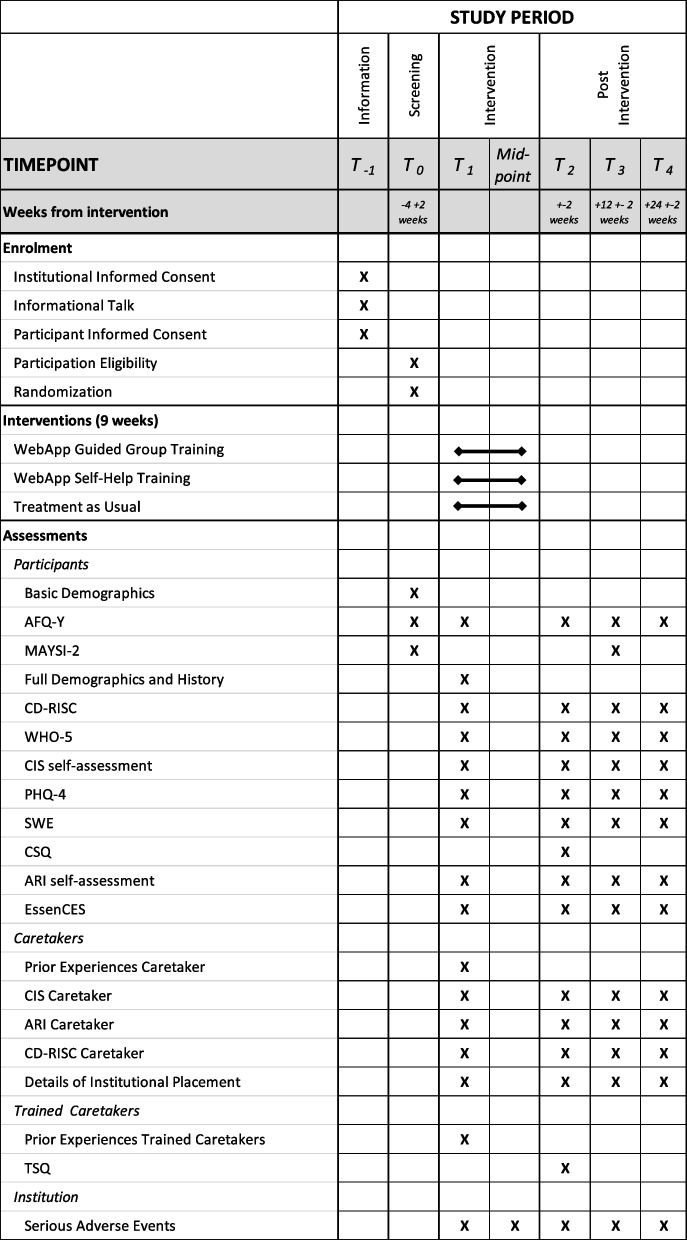
Trial schedule *Note. T*_*1*_,* T*_*2*_,* T*_*3*_,* T*_*4*_ time points; *AFQ-Y* Avoidance and Fusion Questionnaire for Youth; *MAYSI-2* Massachusetts Youth Screening Instrument-2; *CD-RISC* Connor-Davidson Resilience Scale; *WHO-5* World Health Organization – Five Well-Being Index; *CIS* Columbia Impairment Scale; *PHQ-4* Patient Health Questionnaire-4; *SWE* Skala zur Allgemeinen Selbstwirksamkeitserwartung [General Self-Efficacy Expectancy Scale]; *CSQ* Client Satisfaction Questionnaire; *ARI* Affective Reactivity Index; *EssenCES* Essen Climate Evaluation Schema; *TSQ* Trainer Satisfaction Questionnaire*.* Data will be collected at screening (T_0_), ≤ 4 (+ 2) weeks before the intervention (T_1_; baseline assessment), ≤ 2 weeks after the intervention has ended (T_2_; post-intervention assessment), 12 ± 2 weeks post intervention (T_3_; first follow-up), and 24 ± 2 weeks post intervention (T_4_; second follow-up)

Figure 1 provides an overview of the trial schedule, including details on enrolment, interventions, and assessment timelines.

### Sample size {14}

The sample size estimation was performed in statistical software R using a simulation-based approach. The sample size estimation is based on the assumptions, that standard deviations of AFQ-Y scores are equal for all groups and conditions, and that the mean baseline scores of all groups are equal due to randomization. We assumed the AFQ-Y scores at baseline to be normally distributed with a mean of 21.04 and standard deviation of 13.01 [37]. Further, we assumed a high correlation of AFQ-Y scores within participants between time-points, with a Pearson correlation coefficient of *r* ~ = ~ 0.80. The effect sizes of the intervention in standardized Cohen’s *d scores were* = -.50 for TAU vs. web-based self-help training and Cohen's *d* = -.80 for TAU vs. web-based group training.

The above assumptions regarding distribution, correlations, and effect sizes were used to determine the required sample size with a simulation-based approach. Based on the above assumptions and distributions, 9999 synthetic data sets of different sample sizes were generated. The primary analysis was applied to each of these data sets. The simulations showed that at an effective sample of *N* = 78 (26 participants per condition) would enable us to reject the null hypothesis with the desired power of 80%.

**Fig. 2:**
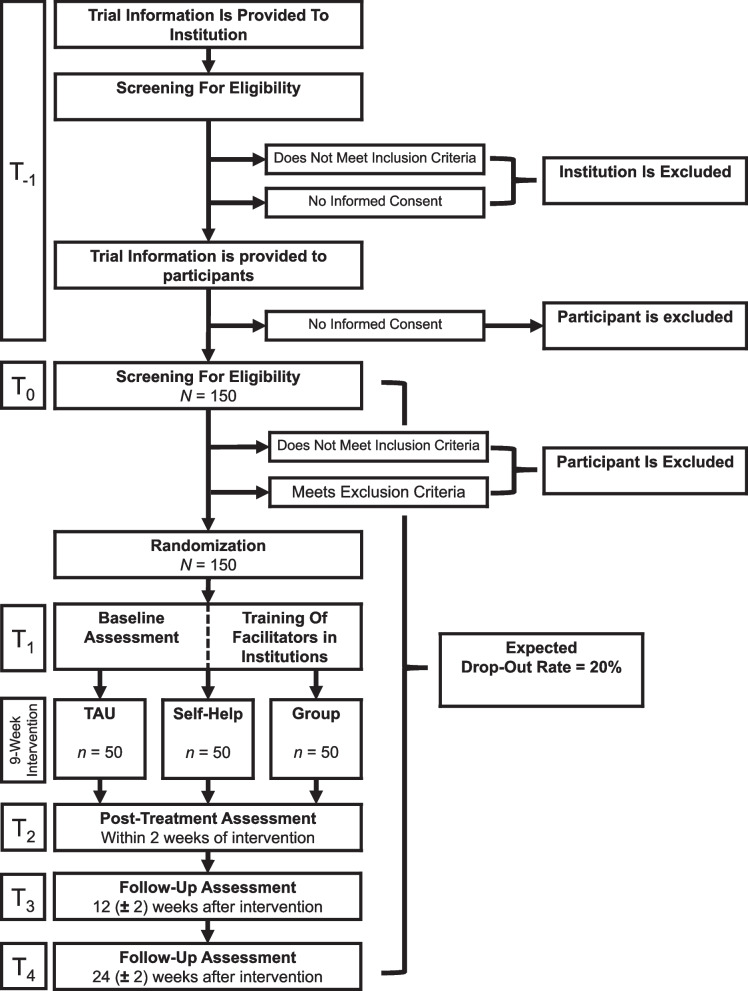
Trial flowchart *Note. TAU* treatment as usual

In the next step, we corrected this sample size for the design effect (i.e., clustering of participants in groups), assuming a weak resemblance in the AFQ-Y score of patients within the same group (rho = 0.2) [53]. Before the start of the study, we assumed a group size (i.e., participants receiving the intervention together) of on average 9 participants. During a sample size re-estimation, this number was reduced to 4 participants per group as was observed in the study centers. With an estimated drop-out rate of 20% [17], 150 patients (i.e., 50 patients per condition) must be recruited to reject our null hypothesis at a power of 80% considering the effect of clustering. The expected flow of participants from recruitment through the end of the study is shown in Fig. 2.

### Recruitment {15}

All institutions recognized by the FOJ will be informed about the study through mail, phone, and announcements by the study team in Basel. Interested and eligible institutions will be provided with recruitment material. Potential participants will be linked to the START NOW Basel study team and received all information about the study during a video call: nature of the study, its purpose, the procedures involved, the expected duration, and potential risks and benefits. Each potential participant will be informed that the participation in the study is voluntary and that he or she may withdraw from the study at any time and that withdrawal of consent will not affect his or her subsequent treatment. Study information and informed consent forms will be provided to all participating institutions.

To ensure thorough recruitment, all recognized institutions will be contacted via various channels, including mail, phone, and personal contact. The trial systematically addresses all eligible participants within the designated timeframe, with ongoing communication efforts. Adolescent participants will receive compensation and retained WebApp access throughout, with TAU participants gaining access post follow-up II. Participating institutions will receive training in START NOW, open to interested staff, and institution in the TAU condition will receive training and WebApp access after follow-up II.

### Assignment of interventions: allocation {16}

#### Sequence generation {16a}

The randomization process will occur at the institutional level when a minimum of four eligible participants were available (gave informed consent and passed screening). Participants will be randomized within fully eligible groups of 4 to 12 individuals, stratified by study site and across 3 conditions. Institutions will be randomized using a pre-generated list, generated using the statistical software R, inaccessible to the research team, and provided and implemented through the DCR at the University of Basel.

#### Concealment mechanism {16b}

The allocation sequence is solely handled by the Clinical Trial Unit of the DCR and is not accessible to the study team. Upon randomization, allocation will be revealed to the study team members via REDCap. Subsequently, the study team members will inform participating institutions and AYAsn.

#### Implementation {16c}

Institutional randomization will occur after obtaining consent from at least four eligible participants within an institution. The process will be initiated using a pre-generated list attached to REDCap, which is not visible to the study team. The allocation sequence is generated by the DCR.

### Assignment of interventions: blinding {17}

#### Who will be blinded {17a}

Not applicable. After randomization, the START NOW study team will inform the participating institution, and consequently, the participants, about their allocation to the respective condition.

#### Procedure for unblinding if needed {17b}

Not applicable, as the study design does not involve blinding.

### Data collection and management {18}

#### Plans for assessment and collection of outcomes {18a}

Data, including electronic Case Report Form (eCRF), will be collected using REDCap. Participants and caretakers will fill out questionnaires, while trained members of the study team will enter objective study data (time-points, participant data from consent form, etc.). Participants will be sent personalized links to the questionnaires at standardized time-points. Questionnaires can be completed on mobile and desktop devices with internet access. WebApp usage data will be collected automatically.

#### Plans to promote participant retention and complete follow-up {18b}

Participants are consistently reminded to complete the questionnaires. As compensation for completing all online questionnaires at survey times T_1_ to T_4_, all participants can receive shopping vouchers worth a maximum of CHF 100.-. For each missing T, CHF 20.- will be deducted from the total. The respective shopping vouchers will be handed out at the end of the participation period.

### Data management {19}

Trial data is stored in REDCap, including online questionnaire data and eCRF. Serious adverse events (SAE) will be recorded in the eCRF. The online platform REDCap is operated in compliance with 21 CFR Part 11 to ensure restricted access for qualified personnel, data security, incident, and change management. Forms validate all entries of the study team, participants, and caretakers. All changes are logged automatically with no option for the study team to manipulate the history.

Informed consent forms will be stored in paper within a locked cabinet in a secured room. WebApp usage data is stored in the WebApp and is only accessible to administrators. All members of the study team are instructed in-person and with videos, with all training sessions and permissions logged. Protocol violations will be recorded separately from study data and provided to the monitor.

### Confidentiality {27}

The principal investigator will maintain a subject ID list to enable records to be identified. The participant identification list will be stored in the Investigator Site File that is locked away in a fire-secure locker in a dedicated room in the research department of Child and Adolescent Psychiatry at UPK. Only authorized staff have access. Authorized personnel may inspect the subject-related data collected during the trial, ensuring compliance with the data protection law (inspectors, monitors, auditors). A back-up digital Excel file will be stored on a laptop with no internet access, located in a dedicated locked room. Only study investigators will have access to the passwords required to boot the laptop and open the Excel file.

All study data are archived for 10 years after study termination or premature termination of the study. Trial data will be archived at the Center for Scientific Computing of the University of Basel and WebApp entries will be archived at Arteria GmbH Basel. After these 10 years, all physical records will be destroyed and the document linking personal details and study codes will be deleted.

### Plans for collection, laboratory evaluation, and storage of biological specimens for genetic or molecular analysis in this trial/future use {33}

Not applicable as no biological specimens were used in this trial.

### Statistical methods {20}

#### Statistical methods for primary and secondary outcomes {20a}

A detailed statistical analysis plan will be written by the study statistician and kept under version control at the DCR. The statistical analysis plan will be finalized before the last visit of the last patient and the final version will be stored before access to the study database is granted.

All outcomes of interest will be described stratified by intervention group at each of the time-points of assessment by mean and standard deviation, median, or median and interquartile range, as appropriate. We will also visualize the time course of the endpoints of interest by intervention group.

The primary objective is to compare the follow-up AFQ-Y score (12 ± weeks after the end of the intervention) between the 3 intervention arms. To this end, we will perform an intention-to-treat analysis, analyzing all participants in the groups they were randomized to, regardless if they actually completed the particular intervention. We will use the imputed full analysis set for the primary analysis. The primary endpoint is the change in AFQ-Y score from baseline (T_1_) to the first post intervention follow-up (T_3_, 12 ± weeks). We will fit a linear mixed-effects model for the primary endpoint post intervention AFQ-Y score. The identifier of the institution group will be included as a random intercept to account for the clustered data structure (i.e., participants in training groups within institutions). The intervention (3 levels with TAU as reference) will be included as a fixed effect of interest, and the baseline AFQ-Y score is included in the model as a covariate. We will examine the distribution of the residuals to assess the model assumptions. The estimate of the fixed effect will be presented together with the corresponding 95% confidence interval and p-value. All analyses will be performed in R version 4.3.1 or higher.

Secondary endpoints involve assessing short-term and long-term intervention effects on diverse outcome measures. Short-term effects, immediately post intervention, will be analyzed using various scales, including CD-RISC, SWE, WHO-5, CIS, PHQ-4, and ARI. Long-term effects, at 12- and 24-week follow-up, will concentrate on the AFQ-Y. Employing a linear mixed effects model, analyses will incorporate a random intercept for the group (i.e., training group within institution), intervention as the fixed effect, and baseline scores as covariates. These exploratory analyses will present estimates with 95% confidence intervals, without correction for multiple testing, emphasizing their interpretative nature as potential signals.

#### Interim analyses {21b}

No interim analysis was planned for this study. Given the low risk of the intervention, we did not expect any safety concerns that should be evaluated by an independent data safety and monitoring committee. Furthermore, we did not consider an adaptive trial design and re-evaluation of the sample size during the trial, or stopping due to futility, due to the short duration of recruitment and time between recruitment and primary endpoint assessment.

#### Methods for additional analyses (e.g., subgroup analyses) {20b}

As a secondary objective, we aim to assess the intervention effect in the subset of compliers (i.e., per protocol analysis). We define a participant as a complier if this participant completed at least 50% of the planned group-based training sessions, or if this participant completed at least 50% of the web-based self-help program. The endpoint is the same as for the primary analysis, and the same analysis will be performed on this subset of the full analysis set.

Furthermore, we plan to perform a sensitivity analysis to assess the effect of the imputations on the outcome of the primary analysis. To this end, we will perform the primary analysis on the available-case data set, including all patients without missing data for the variables used in the primary analysis.

#### Methods in analysis to handle protocol non-adherence and any statistical methods to handle missing data {20c}

The primary analysis will be conducted using the full, imputed analysis set comprising all eligible participants with baseline measures of the focal endpoint and according to the intention-to-treat principles. We also define the per-protocol analysis set comprising all eligible participants with a baseline measure for the focal endpoint and considered compliers according to the definition above.

##### Handling missing data

We will use multiple imputation to address the issue of missing outcome values for both primary and secondary endpoints. Missing values will be imputed using Multivariate Imputation by Chained Equations with relevant baseline values and if available values at other follow-up time-points of the focal endpoint.

### Plans to give access to the full protocol, participant-level data and statistical code {31c}

Trial information is confidential until the final report is submitted to the FOJ. Subsequently, only data from participants who have consented to the independent reuse of their data, beyond the START NOW WebApp trial, may be further employed. The dataset can then be provided by the corresponding author upon reasonable request and in accordance with research collaboration and data transfer guidelines. We plan to publish the code for the main analyses with the corresponding papers as a supplement, upon the journal’s request. If it will not be published as part of the appendix, it will be available upon reasonable request to the corresponding author. The pseudonymized data set will be kept for 10 years in accordance with the Swiss legislation.

### Oversight and monitoring {21}

#### Composition of the coordinating center and trial steering committee {5d}

This is a monocentric study, planned, coordinated, and conducted by the START NOW team of the Child and Adolescent Psychiatric Research Department at the University Psychiatric Clinics Basel. The Principal Investigator oversees the study’s implementation, carried out by the study coordinator and a study team. The study team recruits, obtains informed consent, and ensures the protocol’s compliance in accompanying participating institutions and AYA. The study team holds weekly meetings. The sponsor receives biannual updates on the current status of the study. There is no steering committee or other involved parties.

#### Composition of the data monitoring committee, its role and reporting structure {21a}

Not applicable. Considering the minimal risk associated with both the intervention and the trial, there is no evaluation by an independent data safety and monitoring committee.

#### Adverse event reporting and harms {22}

If a SAE occurs, the research project will be interrupted and (i) the event reported to the principal investigator within 24 h and (ii) the Ethics Committee notified on the circumstances via the *Business Administration System for Ethical Committees* (BASEC) within 7 days according to HRO Art. 21. To ascertain that SAE events are reported without any delay, the Basel study team will contact participating institutions via e-mail at T_1_, in the middle of the intervention phase, at T_2_, at T_3_, and at T_4_.

#### Frequency and plans for auditing trial conduct {23}

The Clinical Trial Unit of the DCR is responsible for data monitoring the study. Throughout the study, three monitoring visits (two interim and one final) are carried out in accordance with an established monitoring plan. The process is completely independent from investigators and the sponsor. For more information refer to the monitoring plan.

#### Plans for communicating important protocol amendments to relevant parties (e.g., trial participants, ethical committees) {25}

All amendments, substantial and non-substantial, will be submitted to the EKNZ and implemented only after obtaining the necessary approval. The study team will undergo training on the revised study protocol and all registries will be updated accordingly. In the event of any changes to the trial, affected participants will be notified, and efforts will be made to obtain their re-consent.

The following substantial amendments were made after the initial registration of the trial to enhance participant engagement and accommodate institutional constraints:

Amendments in June 2022:

- Revised procedures for quality assurance and trainer support.
- Revised exclusion criteria to prevent excessive exclusion of potential participants.
- Introduced participant reimbursement.
- Decreased the minimum number of participants per institution to 4 to enable participation of smaller institutions.
- Adjusted randomization timing to minimize waiting periods, facilitating more efficient planning of trainer and staff training sessions based on randomization conditions.
- Adding email addresses to participant consent to streamline staff workload and align with preferred contact methods.

Amendments in September 2022:

- Intervention phase shortened from 12 to 9 weeks to accommodate varying participant lengths of stay.
- Facilitating participation for adolescents and institutions by allowing multiple consecutive or parallel groups within large institutions (same condition).
- Adjusted sample size to 150 due to smaller-than-expected group sizes in previous recruitment, with calculations based on groups of 4 participants.
- All materials were provided in French for the French-speaking part of Switzerland.

Amendments in August 2023:

- Individual expert interviews instead of group discussions for Trainer Satisfaction to accommodate resource constraints of some institutions and ensure their participation.

### Dissemination policy {31}

#### Dissemination plans {31a}

Trial information is confidential until the final report is submitted to the Swiss Federal Office of Justice. Following the final report, the results of this trial will be disclosed completely in international peer-reviewed journals. All interested participants can receive a layman's summary of the results upon request.

## Discussion

This randomized controlled trial aims to investigate the efficacy of a web-based translation of START NOW, promoting PF in institutionalized AYA and addressing implementation barriers in RYC and CI. Within participating institutions, 150 AYA with a need to improve resilience will be randomly assigned at the institution level to one of three conditions (i) a 9-week web-based group training guided by a facilitator, (ii) a 9-week web-based self-help training, and (iii) TAU.

### Limitations

The trial has several limitations. First, challenges to engage institutions and participants arise from limited resources and the lack of motivation to participate in a study. Secondly, we anticipate a high dropout rate due to AYA who are difficult to reach. The lengthy 9-month duration of the trial, influenced by factors such as holidays and relocations, contributes to this expectation. Thirdly, recruitment difficulties also stem from structural variations among institutions, non-participation due to resource constraints on the institutional side, and scheduling challenges during vacations. Additionally, the extended follow-up periods post intervention constitute a fourth challenge that may impact participant engagement. Lastly, self-help interventions and questionnaire adherence exhibit low levels among participants, contributing to the overall limitations. These challenges underscore the necessity for cautious result interpretation.

### Strengths

This trial promises valuable insights into the efficacy of a web-based intervention. By utilizing both a group guided by a facilitator and self-help as comparator groups, our goal is to investigate the optimal level of support. This approach not only explores the efficacy of the intervention but also provides a more comprehensive understanding of the required support levels. Importantly, the trial ensures universal access to evidence-based training for emotional regulation and resilience among AYA, leveraging innovative technologies for program appeal and resource efficiency. All training materials remain accessible online even after the funding period concludes ensuring sustainability. Moreover, the inclusion of French-speaking cantons facilitates broad dissemination of the WebApp. Beyond its inherent effectiveness, the training incorporates a dissemination strategy that empowers both employees and AYA to become trainers, fostering resilience and perceived self-efficacy within the community.

## Trial status

At the time of manuscript submission, all participants are in the follow-up assessment period (T_3_ to T_4_). Recruitment started in March 2022 and lasted until July 2023. Submitting the study protocol earlier was not feasible due to challenges, including staff turnover in the study core team and DCR, as well as multiple revisions influenced by participating institutions and a second language region. The current protocol is version 5 of 28-08-2023. The trial closure is scheduled for March 2024, following the last participant`s completion.

### Supplementary Information

Supplementary Material 1.

## Data Availability

This study protocol is officially registered with ClinicalTrials.gov (Identifier: NCT05313581; registered on 6 April 2022; last updated on 7 April 2024). Data and materials will be publicly available upon final report to the Swiss Federal Office of Justice.

## Abbreviations

ACT: Acceptance and commitment therapy
AE: Adverse event
AFQ-Y: Avoidance and Fusion Questionnaire for Youth
ARI: Affective Reactivity Index
AYA: Adolescents and young adults
BASEC: Business Administration System for Ethical Committees
CBT: Cognitive behavioral therapy
CD-RISC: Connor-Davidson Resilience Scale
CI: Correctional institutions
CIS: Columbia Impairment Scale
ClinO: Ordinance on Clinical Trials in Human Research
CSQ: Client Satisfaction Questionnaire
DBT: Dialectical behavior therapy
DCR: Department of Clinical Research
EKNZ: Ethikkommission Nordwest- und Zentralschweiz [Ethics Commission Northwestern and Central Switzerland]
eCRF: Electronic Case Report Form
EssenCES: Essen Climate Evaluation Schema
FOJ: Federal Office of Justice
MAYSI-2: Massachusetts Youth Screening Instrument-2
MI: Motivational interviewing
PHQ-4: Patient Health Questionnaire-4
PF: Psychological flexibility
RYC: Residential youth care
SAE: Serious adverse event
SWE: Skala zur Allgemeinen Selbstwirksamkeitserwartung [General Self-Efficacy Expectancy Scale]
TAU: Treatment as usual
TSQ: Trainer Satisfaction Questionnaire
WebApp: Web Application
WHO-5: World Health Organization – Five Well-Being Index
