# Supplementary material for: START NOW WebApp—promoting emotion regulation and resilience in residential youth care and correctional institutions: study protocol for a cluster randomized controlled trial
Source: Trials. 2024 May 22;25:341. doi: 10.1186/s13063-024-08180-z (PMC11112814; doi:10.1186/s13063-024-08180-z)
Supplement: Supplementary file 1 — Supplementary Material 1. [file 13063_2024_8180_MOESM1_ESM.pdf]

UNI BASEL

UPK Universitäre Psychiatrische Kliniken Basel

«START NOW WebAPP: Skilltraining zur Förderung der Resilienz»
(START NOW WebAPP)

Organisiert ist das Projekt durch: Prof. Dr. Dr. Christina Stadler

Sehr geehrte Dame, sehr geehrter Herr
Liebe Jugendliche

Wir möchten Dich fragen, ob Du an unserem Forschungsprojekt teilnehmen möchtest. Im Folgenden erklären wir in einer Zusammenfassung, um was es dabei geht. Du kannst aber auch alles in einer ausführlichen Beschreibung lesen.

Zusammenfassung

**1**	**Ziel des Projekts**<br>Uns interessiert, ob das **Training START NOW**, bei dem eine neu entwickelte WebAPP verwendet wird, hilfreich ist.
**2**	**Auswahl**<br>Du bist zwischen 14 und 24 Jahre alt, lebst in einer Institution, und hast ausreichend Deutsch- oder Französischkenntnisse.
**3**	**Allgemeine Informationen zum Projekt**<br>Im START NOW Training werden Strategien vermittelt und trainiert, um optimal mit Stress umzugehen und auch schwierige Emotionen angemessen regulieren zu können. Menschen, die das können, sind widerstandsfähig (resilient), sie haben eine gute psychische Fitness und fühlen sich wohler.<br>Eine Beschreibung dieser Studie findest Du auch auf der Internetseite des Bundesamtes für Gesundheit unter www.kofam.ch. In diesem Projekt sollen in den nächsten 2 Jahren rund 150 Jugendliche und junge Erwachsene trainiert und befragt werden.
**4**	**Ablauf**<br>Wenn Du einverstanden bist, bei der Studie mitzumachen, überprüfen wir, ob die Kriterien für die Studienteilnahme erfüllt sind. Dann wirst Du zufällig einer der drei Untersuchungsbedingungen zugeordnet: Selbsthilfe, Gruppe oder Wartegruppe. Teilnehmende der Wartegruppe bekommen das Training ebenfalls, aber später.<br>In der Bedingung „Selbsthilfe“ oder „Gruppe“ machst Du während 9 Wochen jede Woche START NOW Sessions mit der APP (insgesamt 12 Sitzungen), entweder allein für Dich oder in der Gruppe. Die Gruppe kann in Deiner Institution stattfinden oder, wenn das nicht möglich ist, bieten wir auch eine online Gruppe an. Zusätzlich gibt es auf der APP Übungen, mit denen Du Deine psychische Fitness trainieren kannst. Während des Projekts kann es sein, dass eine Person aus unserem Forschungsteam einmal an einer Gruppensitzung dabei sein wird.<br><br>Vor dem Training schicken wir Dir online einige Fragebögen. Die Online-Befragung dauert jeweils ungefähr zwischen 45 und 60 Minuten. Um die Wirksamkeit des Trainings zu überprüfen, füllen die Teilnehmenden, die das START NOW Training gemacht haben, aber auch die Teilnehmenden der Wartegruppe einen Teil der Fragebögen nach 9 Wochen nochmals aus und dann nochmals drei und sechs Monate später. Auf diese Weise können wir überprüfen, ob das Training im Vergleich zur der Wartekontrollgruppe (die das Training vorerst nicht bekommen hat) wirklich effektiv ist und auch, ob die Effekte über einen längeren Zeitraum anhalten.<br><br>Falls Du die Kriterien zur Teilnahme nicht erfüllst, erhältst Du trotzdem Zugang zur App. Details hierzu findest Du im ausführlichen Teil.

5	**Nutzen**<br>Mit Deiner Teilnahme leistest Du einen Beitrag zum Verständnis, ob das bereits geprüfte Gruppentraining START NOW mit Hilfe einer APP auch als geleitetes Gruppentraining und als Selbsthilfe-Training hilfreich ist. Es kann aber auch sein, dass die Teilnahme keinen Nutzen mit sich bringt.
6	**Rechte**<br>Du entscheidest freiwillig, ob Du an diesem Projekt teilnehmen willst oder nicht. Deine Entscheidung hat keinen Einfluss auf etwaige medizinische Behandlung / Betreuung und Du musst diese Entscheidung nicht begründen.
7	**Pflichten**<br>Wenn Du teilnimmst, bitten wir Dich, alle Fragen der Online-Befragung wahrheitsgemäss, nach bestem Wissen und Gewissen, zu beantworten.
8	**Risiken**<br>Eine Teilnahme an dieser Studie ist mit keinerlei Risiken verbunden.
9	**Ergebnisse**<br>Bei neuen Ergebnissen während des Projekts wirst Du informiert. Die Projektleitung kann Dir am Ende der Studie eine Zusammenfassung der Gesamtergebnisse schicken.
10	**Vertraulichkeit von Daten**<br>Wir erheben Deine persönlichen und medizinischen Daten. Die Daten werden in verschlüsselter Form weiterverwendet. Wir halten alle gesetzlichen Regeln des Datenschutzes ein. Alle Beteiligten unterliegen der Schweigepflicht.
11	**Rücktritt**<br>Du kannst jederzeit von dem Projekt zurücktreten und nicht mehr teilnehmen. Die bis dahin erhobenen Daten werden noch ausgewertet.
12	**Entschädigung**<br>Wir stellen Dir die APP kostenlos zur Verfügung. Bisherige Studien haben gezeigt, dass START NOW eine sehr wirksame Behandlung ist, um soziale Kompetenzen zu verbessern.<br>**Für das Ausfüllen der Online-Fragebögen (alle 4 Zeitpunkte) kriegst Du Einkaufsgutscheine im Wert von max. CHF 100.-. Die Einkaufsgutscheine werden am Ende der Teilnahme verteilt.**
13	**Haftung**<br>Die Haftpflichtversicherung der UPK kommt für allfällige Schäden im Rahmen des Projekts auf.
14	**Finanzierung**<br>Das Projekt wird vom Bundesamt für Justiz und der Forschungsabteilung der Klinik für Kinder und Jugendliche der UPK finanziert.
15	**Kontaktperson**<br>Du kannst jederzeit auf alle Deine Fragen Auskunft erhalten.<br><br>Projektleitung: Prof. Dr. Dr. Christina Stadler<br>Klinische Professorin für Entwicklungspsychopathologie<br>Universitäre Psychiatrische Kliniken (UPK) Basel<br>Klinik für Kinder und Jugendliche<br>Forschungsabteilung<br>Wilhelm-Klein-Strasse 27<br>CH-4002 Basel<br>Email: christina.stadler@upk.ch<br>Tel : +41 61 325 82 79

Mit Deiner Unterschrift am Ende des Dokuments bezeugst Du, dass Du freiwillig teilnimmst und dass Du die Inhalte des gesamten Dokuments verstanden hast.

Detailliertere Information

1. Ziel des Projekts

Uns interessiert, ob das Training START NOW auch als WebAPP hilfreich ist. Konkret wollen wir die Frage untersuchen, ob man seine psychische Fitness steigern kann, wenn man die APP allein als reines Selbsthilfetraining durchführt oder ob man bessere Effekte erzielt, wenn man bei der

Durchführung Unterstützung durch einen Trainer in einer Gruppe erhält. Uns interessiert ausserdem, ob Verbesserungen über einen längeren Zeitraum anhalten.
Onlinefragebögen helfen uns dabei Veränderungen, aber auch Deine Zufriedenheit mit dem Trainingsprogramm zu erfassen. All dies hilft uns dabei zu überprüfen, ob und wie die APP in Zukunft eingesetzt werden könnte.

2. Auswahl

Du bist zwischen 14 und 24 Jahre alt, lebst in einer Institution, und hast ausreichend Deutsch- oder Französischkenntnisse. Am Anfang prüfen wir mit zwei Onlinefragebögen, ob Du unsere Einschlusskriterien, psychische Fitness verbessern und keine akute psychische Belastung, erfüllst. Falls du unsere Einschlusskriterien nicht erfüllst, weil du schon Strategien im Umgang mit Herausforderungen hast, kannst du trotzdem mitmachen und hoffentlich Neues lernen.

3. Allgemeine Informationen zum Projekt

Fast jeder junge Mensch hat in seinem Leben schon schwierige Situationen, Krisen und Stress erlebt. Im START NOW Training werden Strategien vermittelt und trainiert, um optimal mit Stress umzugehen und auch schwierige Emotionen angemessen regulieren zu können. Menschen, die das können, sind widerstandsfähig (resilient), sie haben eine gute psychische Fitness und fühlen sich wohler.
Das START NOW Training ist wirksam, das haben vorherige Studien gezeigt, aber bisher gab es keine APP. Wir gehen davon aus, dass eine APP für Jugendliche und junge Erwachsenen attraktiv ist und zusätzliche Vorteile bringen kann. Deshalb untersuchen wir in diesem Projekt, wie die APP am besten eingesetzt werden kann: Gibt es bessere Effekte, wenn man durch einen Trainer unterstützt wird oder verbessert sich die psychische Fitness (Widerstandsfähigkeit) auch im Selbsthilfe-Ansatz? Um die Wirksamkeit wissenschaftlich testen zu können, müssen wir auch eine Gruppe untersuchen, die das Training nicht (bzw. später) bekommt, das ist die Wartegruppe.
Onlinefragebögen helfen uns dabei Veränderungen und Effekte zu erfassen.
Eine Beschreibung dieser Studie findest Du auch auf der Internetseite des Bundesamtes für Gesundheit unter www.kofam.ch. In diesem Projekt sollen in den nächsten 2 Jahren rund 150 Jugendliche und junge Erwachsene trainiert und befragt werden.
Dieses Projekt wurde so erstellt, wie es die Gesetze in der Schweiz vorschreiben. Die zuständige Ethikkommission hat unsere Studie geprüft und bewilligt.

4. Ablauf

Wenn Du einverstanden bist, bei der Studie mitzumachen, überprüfen wir, ob die Kriterien für die Studienteilnahme erfüllt sind (max. 45 Minuten). Dann wirst Du zufällig einer der drei Untersuchungsbedingungen zugeordnet: Selbsthilfe, Gruppe oder Wartegruppe. Teilnehmende der Wartegruppe bekommen das Training ebenfalls, aber erst nach 9 Monaten. Falls Du vor Beginn des Trainings die Institution verlassen solltest, stellen wir Dir die APP mit allen Materialien kostenlos zur Verfügung, um Dir zu ermöglichen, die verschiedenen START NOW Strategien auf alle Fälle kennenzulernen.
In der Bedingung „Selbsthilfe“ oder „Gruppe“ machst Du während 9 Wochen jede Woche START NOW Sessions mit der APP (insgesamt 12 Sitzungen; Sitzung 1+2, 9+10 und 11+12 sind Doppelsitzungen), entweder allein für Dich (45 Mintuen, +/- 15 Minuten; Doppelsitzungen 90, +/-15 Minuten) oder in der Gruppe (60 Minuten; Doppelsitzungen 120 Minuten). Die Gruppe kann in Deiner Institution stattfinden (Trainer ist Institutionsmitarbeitender) oder, wenn das nicht möglich ist, bieten wir auch eine online Gruppe an (externe Personen). Zusätzlich gibt es auf der APP Übungen, mit denen Du Deine psychische Fitness trainieren kannst. In den Sitzungen geht es unter anderem auch um den Umgang mit Emotionen, Achtsamkeitsübungen und eigene Ziele.

Für das Online-Training verwenden wir Zoom (Zoom Video Communications, Inc., San Jose Headquarters, 55 Almaden Boulevard, Suite 600, San Jose, CA 95113, USA) einen Videokonferenzdienst, mit dem man sich virtuell mit anderen treffen kann.

Alle START NOW Trainer werden vor der Durchführung von uns ausgebildet, wie sie das Training durchführen. Während des Projekts kann es sein, dass eine Person aus unserem Forschungsteam einmal an einer Gruppensitzung dabei sein wird, um zu prüfen ob das Training korrekt durchgeführt wird.

Vor dem Training schicken wir Dir online einige Fragebögen. Die Online-Befragung dauert jeweils ungefähr zwischen 45und 60 Minuten. In diesen geht es darum wie Du mit verschiedenen Situationen umgehst, wie Du Dein eigenes Verhalten einschätzt und wie Du dein Umfeld und andere wahrnimmst. Um die Wirksamkeit des Trainings zu überprüfen, füllen die Teilnehmenden, die das START NOW Training gemacht haben, aber auch die Teilnehmenden der Wartegruppe einen Teil der Fragebögen nach 9 Wochen nochmals aus und dann nochmals drei und sechs Monate später. Auf diese Weise können wir überprüfen, ob das Training im Vergleich zur der Wartekontrollgruppe (die das Training vorerst nicht bekommen hat) wirklich effektiv ist und auch, ob die Effekte über einen längeren Zeitraum anhalten.

Falls Du die Kriterien zur Teilnahme nicht erfüllst, erhältst Du trotzdem Zugang zur App und wirst jede Woche zu einer neuen Session freigeschaltet (ähnlich der Selbsthilfe Bedingung). Auch hier nimmst Du an den gleichen Online-Befragungen vor dem Training teil, wir schicken Dir nach Abschluss noch einen Fragebogen zu Deiner Zufriedenheit mit dem Training. Danach werden keine Befragungen mehr durchgeführt. Mit Deinen Daten werden wir zum Beispiel prüfen, ob die verwendeten Fragebögen auch das messen was sie messen sollen und wie wir das Training verbessern können.

5. Nutzen

Mit Deiner Teilnahme leistest Du einen Beitrag zum Verständnis, ob das bereits geprüfte Gruppentraining START NOW mit Hilfe einer APP auch als geleitetes Gruppentraining und als Selbsthilfe-Training hilfreich ist. Es kann aber auch sein, dass die Teilnahme keinen Nutzen mit sich bringt.

6. Rechte

Du entscheidest freiwillig, ob Du an diesem Projekt teilnehmen willst oder nicht. Wenn Du nicht teilnehmen oder später Deine Teilnahme zurückziehen willst, musst Du dies nicht begründen. Deine medizinische Behandlung/Betreuung ist unabhängig von Deinem Entscheid sichergestellt. Du darfst jederzeit Fragen zur Teilnahme und zum Projekt stellen. Wende Dich dazu bitte an die Person, die am Ende dieser Information angegeben ist.

7. Pflichten

Wenn Du teilnimmst, bitten wir Dich, alle Fragen der Online-Befragung wahrheitsgemäss, nach bestem Wissen und Gewissen, zu beantworten.

8. Risiken

Eine Teilnahme an dieser Studie ist mit keinerlei Risiken verbunden. Das Ausfüllen der Fragebögen kann höchstens dann ein geringfügiges Risiko darstellen, wenn es zu der gedanklichen Beschäftigung mit möglicherweise schwierigen und als belastend empfundenen Ereignissen aus der Vergangenheit kommt. Sollte dies bei Dir zu einer Beeinträchtigung führen, nimm bitte Kontakt mit Deiner Bezugsbetreuerin/Deinem Bezugsbetreuer auf. Ausserdem findest Du in der APP einen Hilfe-Button mit Notfall- und Beratungsadressen.
Bitte Nutze die APP über eine kostenlose W-LAN Verbindung, damit für dich keine Zusatzkosten entstehen.

9. Ergebnisse

Die Prüfperson/Projektleitung wird Dich während des Projekts über alle neuen Erkenntnisse informieren, die den Nutzen oder Deine Sicherheit und somit Deine Einwilligung zur Teilnahme beeinflussen können. Du wirst schriftlich informiert und kannst dann erneut entscheiden, ob Du

weiter an der Studie teilnehmen möchtest. Die Projektleitung kann Dir am Ende der Studie eine Zusammenfassung der Gesamtergebnisse schicken.

10. Vertraulichkeit von Daten

Wir erheben Deine persönlichen und medizinischen Daten. Die Daten werden in verschlüsselter Form weiterverwendet. Wir halten alle gesetzlichen Regeln des Datenschutzes ein. Alle Beteiligten unterliegen der Schweigepflicht. Nur sehr wenige Fachpersonen werden Deine unverschlüsselten Daten sehen, um Aufgaben im Rahmen des Projekts zu erfüllen. Diese Verschlüsselung bedeutet, dass alle Bezugsdaten, die die Teilnehmenden identifizieren könnten (Name, Geburtsjahr), gelöscht und durch einen Schlüssel (Code) ersetzt werden. Diejenigen Personen, die den Schlüssel nicht kennen, können daher keine Rückschlüsse auf die Teilnehmenden ziehen. Die Schlüssel-Liste bleibt immer in den Gebäuden der UPK in einem gesicherten Schrank. Bei einer Publikation sind die zusammengefassten Daten daher auch nicht auf Dich als Einzelperson zurück zu verfolgen. Dein Name taucht niemals im Internet oder einer Publikation auf. Manchmal gibt es die Vorgabe bei einer Zeitschrift zur Publikation, dass Einzel-Daten (sogenannte Roh-Daten) übermittelt werden müssen. Wenn Einzel-Daten übermittelt werden müssen, dann sind die Daten immer verschlüsselt und somit ebenfalls nicht auf Dich als Person zurück zu verfolgen. Alle Personen, die im Rahmen des Projekts Einsicht in Deine Daten haben, unterliegen der Schweigepflicht. Die Vorgaben des Datenschutzes werden streng eingehalten und Du als teilnehmende Person hast jederzeit das Recht auf Einsicht in Deine Daten.

Es ist möglich, dass Deine Daten für andere Projekte zu einem späteren Zeitpunkt weiterverwendet werden. Diese andere Datenbank muss die gleichen Standards einhalten wie die Datenbank zu diesem Projekt. Für diese Weiterverwendung bitten wir Dich, ganz am Ende dieses Dokuments eine weitere Einwilligungserklärung zu unterzeichnen. Diese zweite Einwilligung ist unabhängig von der Teilnahme an dieser Studie.

Möglicherweise wird dieses Projekt durch die zuständige Ethikkommission oder durch die Institution, die das Projekt veranlasst hat, überprüft. Die Prüfperson/die Projektleitung muss dann Deine Daten für solche Kontrollen offenlegen. Alle müssen absolute Vertraulichkeit wahren.

11. Rücktritt

Du kannst jederzeit von dem Projekt zurücktreten und nicht mehr teilnehmen. Die bis dahin erhobenen Daten werden noch ausgewertet. Nach der Auswertung werden Deine Daten anonymisiert, d.h. die Schlüsselzuordnung wird vernichtet, so dass danach niemand mehr erfahren kann, dass die Daten ursprünglich von Dir stammten (Datenschutz).

12. Entschädigung

Wir stellen Dir die APP kostenlos zur Verfügung. Bisherige Studien haben gezeigt, dass START NOW eine sehr wirksame Behandlung ist, um soziale Kompetenzen zu verbessern.
Für das Ausfüllen aller Online-Fragebögen zu den Zeitpunkten T1 bis und mit T4 (4 Zeitpunkte) kriegst Du Einkaufsgutscheine im Wert von max. CHF 100.-. Für jeden fehlenden Zeitpunkt T werden jeweils CHF 20.- abgezogen. Die Einkaufsgutscheine werden am Ende der Teilnahme verteilt.

13. Haftung

Falls Du durch das Projekt einen Schaden erleidest, haftet die UPK, die das Projekt veranlasst hat und für die Durchführung verantwortlich ist. Die Voraussetzungen und das Vorgehen sind gesetzlich geregelt.

14. Finanzierung

Das Projekt wird vollständig vom Bundesamt für Justiz und der Forschungsabteilung der Klinik für Kinder und Jugendliche der UPK finanziert.

15. Kontaktperson(en)

Du darfst jederzeit Fragen zur Studienteilnahme stellen. Auch bei Unsicherheiten oder Notfällen, die während der Studie oder danach auftreten, wende Dich bitte an:

Projektleitung: Prof. Dr. Dr. Christina Stadler
Klinische Professorin für Entwicklungspsychopathologie
Universitäre Psychiatrische Kliniken (UPK) Basel
Klinik für Kinder und Jugendliche
Forschungsabteilung
Wilhelm-Klein-Strasse 27
CH-4002 Basel
Christina.Stadler@upk.ch
Tel +41 61 325 82 79

Projektkoordination: Dr. Donja Brunner
Donja.Brunner@upk.ch
Tel +41 61 325 80 31

Einwilligungserklärung

Schriftliche Einwilligungserklärung zur Teilnahme an einer klinischen Studie
Bitte lies dieses Formular sorgfältig durch. Bitte frag nach, wenn Du
Du etwas nicht verstehst oder wissen möchtest. Für die Teilnahme ist Deine schriftliche Einwilligung notwendig.

**BASEC-Nummer (nach Einreichung):**	2022-00108
**Titel der Studie (wissenschaftlich und Laiensprache)**:	**„START NOW: START NOW WebAPP: Skilltraining zur Förderung der Resilienz“**<br><br>Implementierung einer e-Version des Skilltrainings START NOW zur Förderung der Emotionsregulation und Resilienz in sozialpädagogischen Institutionen und Institutionen des Massnahmenvollzugs<br><br>(START NOW WebAPP)
**Verantwortliche Institution (Sponsor mit Adresse**):	Projektleitung: Prof. Dr. Dr. Christina Stadler<br>Klinische Professorin für Entwicklungspsychopathologie<br><br>Universitäre Psychiatrische Kliniken (UPK)<br>Klinik für Kinder und Jugendliche<br>Forschungsabteilung<br>Wilhelm-Klein-Strasse 27<br>CH-4002 Basel<br>Email: christina.stadler@upk.ch<br>Tel : +41 61 325 82 79
**Ort der Durchführung**:	UPK Basel
**Prüfperson am Studienort:**<br>Name und Vorname in Druckbuchstaben:	Prof. Dr. Dr. Stadler, Christina
**Teilnehmerin/Teilnehmer:**<br>Name und Vorname in Druckbuchstaben:	
Geburtsdatum:	
E-Mail-Adresse:	

- Ich wurde von der unterzeichnenden Prüfperson mündlich und schriftlich über den Zweck, den Ablauf der Studie mit dem web-basierten START NOW Training über mögliche Vor- und Nachteile sowie über eventuelle Risiken informiert.
- Ich nehme an dieser Studie freiwillig teil und akzeptiere den Inhalt der mir ausgehändigten schriftlichen Information. Ich hatte genügend Zeit, meine Entscheidung zu treffen.

- Meine Fragen im Zusammenhang mit der Teilnahme an dieser Studie sind mir beantwortet worden. Ich behalte die schriftliche Information und erhalte eine Kopie meiner schriftlichen Einwilligungserklärung.
- Ich bin einverstanden, dass die zuständigen Fachleute der Projektleitung, des Sponsors, der zuständigen Ethikkommission zu Prüf- und Kontrollzwecken in meine unverschlüsselten Daten Einsicht nehmen dürfen, jedoch unter strikter Einhaltung der Vertraulichkeit.
- Ich weiss, dass meine gesundheitsbezogenen und persönlichen Daten nur in verschlüsselter Form zu Forschungszwecken **für diese Studie** weitergegeben werden können. Der Sponsor gewährleistet, dass der Datenschutz nach Schweizer Standard eingehalten wird.
- Ich weiss, dass mein Name in keiner Weise in Rapporten oder Publikationen, die aus der Studie hervorgehen, veröffentlicht wird.
- Ich kann jederzeit und ohne Angabe von Gründen von der Studienteilnahme zurücktreten, ohne dass ich deswegen Nachteile bei etwaiger weiteren medizinischen Behandlung/ Betreuung habe. Die bis zum Rücktritt erhobenen Daten werden noch im Rahmen der Studie ausgewertet.
- Die Haftpflichtversicherung der UPK kommt für allfällige Schäden auf.
- Ich bin mir bewusst, dass die in der Teilnehmerinformation genannten Pflichten einzuhalten sind. Im Interesse meiner Gesundheit kann mich die Prüfperson/die Projektleitung jederzeit von der Studie ausschliessen.

Ort, Datum	Unterschrift Teilnehmerin/Teilnehmer

Bestätigung der Prüfperson: Hiermit bestätige ich, dass ich dieser Teilnehmerin/diesem Teilnehmer Wesen, Bedeutung und Tragweite der Studie erläutert habe. Ich versichere, alle im Zusammenhang mit dieser Studie stehenden Verpflichtungen gemäss in der Schweiz geltenden Rechts zu erfüllen. Sollte ich im Verlauf der Studie von Aspekten erfahren, welche die Bereitschaft der Teilnehmerin/des Teilnehmers zur Studienteilnahme beeinflussen könnten, werde ich sie/ihn umgehend darüber informieren.

Ort, Datum	Name und Vorname der Prüfperson in Druckbuchstaben<br><br>Unterschrift der Prüfperson

(optional): Einwilligungserklärung für Weiterverwendung von Daten in verschlüsselter Form (für die Weiterverwendung von Daten des Modellversuch Projekts) (online)

**BASEC-Nummer (nach Einreichung):**	2022-00108
**Titel der Studie (wissenschaftlich und Laiensprache)**:	**„START NOW: START NOW WebAPP: Skilltraining zur Förderung der Resilienz“**<br><br>Implementierung einer e-Version des Skilltrainings START NOW zur Förderung der Emotionsregulation und Resilienz in sozialpädagogischen Institutionen und Institutionen des Massnahmenvollzugs<br><br>(START NOW WebAPP)
**Teilnehmerin/Teilnehmer:**<br>Name und Vorname in Druckbuchstaben:	
Geburtsdatum:	

Ich erlaube, dass meine verschlüsselten Daten aus diesem Projekt für die medizinische Forschung weiterverwendet werden dürfen. Diese Einwilligung gilt unbegrenzt.

Ich habe verstanden, dass die Daten verschlüsselt sind und der Schüssel sicher aufbewahrt wird. Die Daten können im In- und Ausland an andere Datenbanken zur Analyse gesendet werden, wenn diese dieselben Standards wie in der Schweiz einhalten. Alle rechtlichen Vorgaben zum Datenschutz werden eingehalten.

Ich entscheide freiwillig und kann diesen Entscheid zu jedem Zeitpunkt wieder zurücknehmen. Wenn ich zurücktrete, werden meine Daten anonymisiert. Ich informiere lediglich meine Prüfperson/die Projektleitung und muss diesen Entscheid nicht begründen.

Normalerweise werden alle Daten gesamthaft ausgewertet und die Ergebnisse zusammenfassend publiziert. Sollte sich ein für mich relevantes Ergebnis ergeben, ist es möglich, dass ich kontaktiert werde. Wenn ich das nicht wünsche, teile ich dies meiner Prüfperson/der Projektleitung mit.

Wenn Ergebnisse aus den Daten kommerzialisiert werden, habe ich keinen Anspruch auf Anteil an der kommerziellen Nutzung.

Ort, Datum	Unterschrift Teilnehmerin/Teilnehmer

Bestätigung der Prüfperson: Hiermit bestätige ich, dass ich dieser Teilnehmerin/diesem Teilnehmer Wesen, Bedeutung und Tragweite der Weiterverwendung von Daten erläutert habe.

Ort, Datum	Name und Vorname der der Prüfperson in Druckbuchstaben<br><br>Unterschrift der der Prüfperson
